# Proprotein Convertase Subtilisin/Kexin Type 9 (PCSK9) Is Not Induced in Artificial Human Inflammation and Is Not Correlated with Inflammatory Response

**DOI:** 10.1128/IAI.00842-19

**Published:** 2020-02-20

**Authors:** Matthias Wolfgang Heinzl, Michael Resl, Carmen Klammer, Margot Egger, Benjamin Dieplinger, Martin Clodi

**Affiliations:** a Department of Internal Medicine, Konventhospital Barmherzige Brueder Linz (St. John of God Hospital Linz), Linz, Austria; b Department of Laboratory Medicine, Konventhospital Barmherzige Brueder Linz (St. John of God Hospital Linz), Linz, Austria; c ICMR–Institute for Cardiovascular and Metabolic Research, JKU Linz, Linz, Austria; University of California San Diego School of Medicine

**Keywords:** proprotein convertase subtilisin/kexin type 9, PCSK9, pathogen lipids, low-density lipoprotein, LDL, inflammation, lipopolysaccharide, LPS, human endotoxin model

## Abstract

Lipoproteins, as well as proprotein convertase subtilisin/kexin type 9 (PCSK9), have been shown to play a key role in the innate immune response. However, knowledge about the role and kinetics of PCSK9 in human inflammation is currently insufficient. This study aimed to investigate the interaction between inflammation and lipid metabolism, including the possible role of PCSK9. A single-blinded, placebo-controlled cross-over study using the human endotoxin model was performed. Ten healthy men received lipopolysaccharide (LPS) or placebo on two different study days after overnight fasting. Lipoproteins as well as PCSK9 were measured repetitively over 48 h. PCSK9 plasma concentrations were not induced by LPS infusion, and no correlation between PCSK9 plasma concentrations and the degree of inflammation could be identified. The observed low-density lipoprotein (LDL) response to inflammation was more complex than anticipated, especially in the very early phase after the inflammatory stimulus. Baseline concentrations of LDL, as well as high-density lipoprotein (HDL), correlated negatively with inflammatory response. Our data suggest that the lipoprotein response to inflammation is independent of PCSK9. The proposed elevations of PCSK9 and suspected correlations between PCSK9 levels and inflammatory response are not supported by our data. (This study has been registered at ClinicalTrials.gov under registration no. NCT03392701.)

## Abstract (precis)

Lipoproteins, as well as proprotein convertase subtilisin/kexin type 9 (PCSK9), have been shown to play a key role in the innate immune response. However, knowledge about the role and kinetics of PCSK9 in human inflammation is currently insufficient. This study aimed to investigate the interaction between inflammation and lipid metabolism, including the possible role of PCSK9. A single-blinded, placebo-controlled cross-over study using the human endotoxin model was performed.

## INTRODUCTION

Interactions between inflammation and lipid metabolism have widely been recognized. Plasma cholesterol behaves as a negative acute-phase reactant, decreasing after surgery, trauma, liver dysfunction, acute hemorrhage, and sepsis (1–4). The extent of hypocholesterolemia is a marker of severity of illness and poor prognosis in critically ill patients (5, 6).

Under inflammatory conditions, lipoproteins seem to exert an important role in the binding and processing of bacterial endotoxins. Clearance of pathogen lipids such as lipopolysaccharide (LPS) from the circulation usually occurs through hepatic Kupffer cells and other macrophages. During this process, these cells are activated and secrete proinflammatory cytokines such as tumor necrosis factor alpha (TNF-α) (7). Alternatively, LPS can be bound by transfer proteins before being incorporated in high-density lipoprotein (HDL) and, subsequently, very-low-density lipoprotein (VLDL) and low-density lipoprotein (LDL) (8, 9). When bound to lipoproteins, LPS seems to be eliminated mostly by hepatocytes and subsequent biliary excretion. In animal models, the neutralization of endotoxin by lipoproteins has been shown to be protective against hypotension, fever, and death (7, 10). Furthermore, infusion of lipoproteins improved survival in sepsis models (11). Clearance of LPS via this lipoprotein-dependent pathway seems to be beneficial in infection due to more rapid clearance of endotoxin and reduced proinflammatory immune response (7, 10).

Proprotein convertase subtilisin/kexin type 9 (PCSK9) is a serine protease that is produced in the liver and secreted into the plasma. It plays a major role in regulating LDL cholesterol by binding to hepatic LDL receptors and promoting their degeneration. Pharmacological inhibition of PCSK9 has been shown to lower LDL cholesterol levels as well as reduce cardiovascular risk (12).

In animal models, PCSK9 has been shown to be a crucial factor in the pathogenesis of LPS-induced inflammation. PCSK9 plasma concentrations are increased under inflammatory circumstances in murine *in vivo* as well as *in vitro* studies (13, 14). PCSK9 knockout mice show considerably lower levels of proinflammatory cytokines, such as TNF-α, interleukin-6 (IL-6), IL-8, and IL-10, following LPS administration and were shown to eliminate LPS much more rapidly than controls. This reduced clearance of LPS under the influence of PCSK9 seems to be due to a lower uptake of LPS by human hepatocytes (10). Furthermore, the pharmacological inhibition of PCSK9 using monoclonal anti-PCSK9 antibodies in a murine sepsis model also leads to lower plasma concentrations of inflammatory cytokines and, importantly, significantly increased survival rates (10).

In clinical studies, PCSK9 plasma concentrations were markedly elevated in trauma as well as in sepsis patients, and significant associations with severity of illness and organ failure have been shown (15, 16).

Administration of Gram-negative bacterial lipopolysaccharide has been used as a model of inflammation and infection in humans and has been shown to reliably induce a febrile systemic inflammatory response (17–22). This human endotoxin model is the most widely used model to study the pathophysiology of systemic inflammation in humans (23).

In summary, PCSK9 seems to play an important role in inflammation (10). However, knowledge about the role and kinetics of PCSK9 in human inflammation and, importantly, the potential benefits of its pharmacological inhibition in infection is still scarce. The aim of this study was to evaluate the effect of human inflammation on lipid metabolism and PCSK9 using the human endotoxin model in healthy volunteers.

## RESULTS

### Proband characteristics.

All probands were recruited from December 2017 until June 2018. Altogether, 24 volunteers were screened, of which 6 were excluded after screening and 8 chose not to participate. The mean age was 24.1 (standard deviation [SD], 3.7) years, and the average body mass index was 25.2 (SD, 1.6) kg/m^2^.

### Inflammation after LPS infusion.

As expected, all subjects except one experienced flu-like symptoms like chills, myalgia, and headache after LPS infusion (23). The peak of symptoms was observed between 60 min and 90 min after infusion. After 300 min, the vast majority of symptoms had abated in all probands. No subject experienced symptoms of any importance after the administration of placebo.

**FIG 1:**
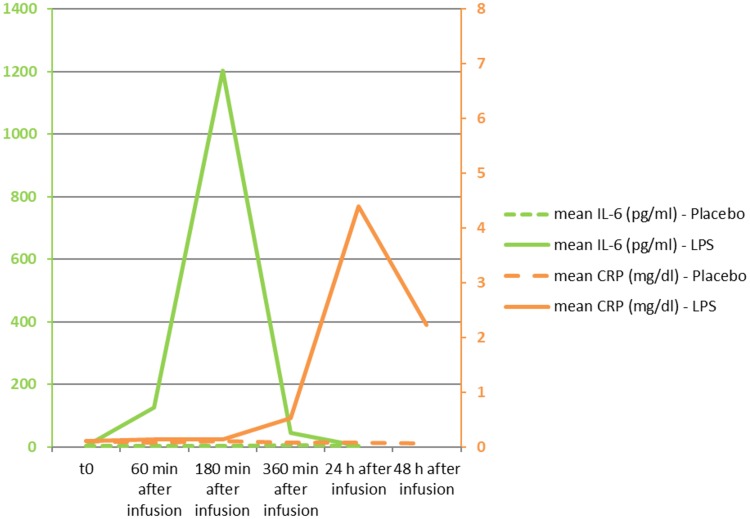
Mean plasma concentrations of interleukin-6 (IL-6) and C-reactive protein (CRP). The difference between placebo and LPS administration was statistically significant for IL-6 (*P* = 0.018) as well as CRP (*P* < 0.001), as measured by RM-ANOVA. IL-6 values are given in picograms per milliliter on the lefthand side; the assay’s upper limit of normal is 15 pg/ml. CRP values are given in milligrams per deciliter on the righthand side; the assay’s upper limit of normal is 1.0 mg/dl.

IL-6, a rapid marker of inflammation, was markedly elevated in all subjects following the administration of LPS. Peak levels were observed at 180 min after infusion. The difference in IL-6 plasma concentrations between LPS infusion and placebo was statistically significant, as analyzed in a repeated-measures analysis of variance (RM-ANOVA) (*P* = 0.018) (Fig. 1). Similarly, C-reactive protein (CRP) plasma concentrations were also significantly induced following LPS administration (*P* < 0.001 as analyzed in an RM-ANOVA) (Fig. 1).

### PCSK9 response after LPS infusion.

**FIG 2:**
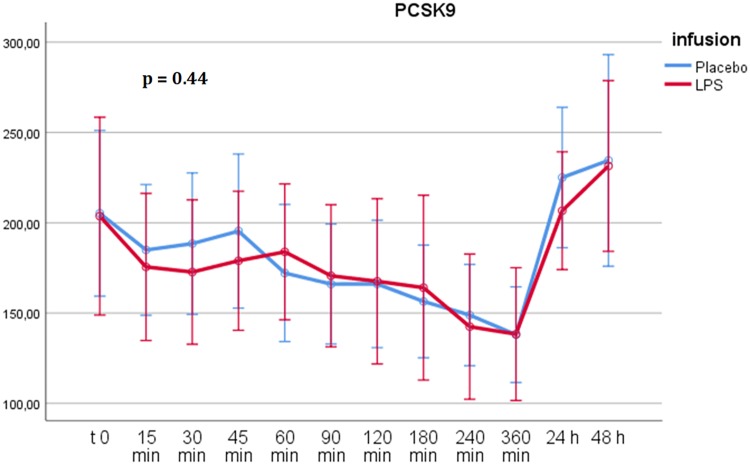
RM-ANOVA for PCSK9. There was no statistically significant difference in PCSK9 plasma concentrations between placebo and LPS administration (*P* = 0.44 using the Greenhouse-Geisser correction). Time points are shown on the abscissa, and plasma concentrations of PCSK9 are shown on the ordinate (values given in nanograms per milliliter). Error bars depict 95% confidence intervals. The decrease of PCSK9 throughout the study day is due to fasting conditions and diurnal variation (26).

After the infusion of both LPS and placebo, PCSK9 plasma concentrations decreased over the study day in all participants. Overall, there was no significant difference in PCSK9 concentrations between the administration of LPS and placebo, as calculated by RM-ANOVA over 12 points in time (*P* = 0.44) (Fig. 2). PCSK9 plasma concentrations did not differ significantly from those of the placebo at any point after LPS infusion.

### Impact of PCSK9 plasma concentrations on inflammatory response.

There was no significant correlation between PCSK9 plasma concentrations at baseline and levels of IL-6 after LPS infusion (Table 1), and there was not a significant correlation between the course of PCSK9 levels after LPS infusion and peak IL-6 plasma concentrations after LPS infusion. Similarly, no significant correlation between PCSK9 plasma concentrations and CRP elevation could be identified.

**TABLE 1:** Correlations between lipid parameters at baseline and IL-6 as a marker of inflammation after LPS infusion^*b*^

Parameter^*a*^	Value for:	
	IL-6 360 min after LPS	IL-6 24 h after LPS
PCSK9 at baseline		
Pearson correlation	−0.326	−0.181
*P* level	0.358	0.618
HDL at baseline		
Pearson correlation	−0.682	−0.793
*P* level	0.030	0.006
ApoA1 at baseline		
Pearson correlation	−0.783	−0.803
*P* level	0.007	0.005
LDL at baseline		
Pearson correlation	−0.494	−0.699
*P* level	0.147	0.024
ApoB at baseline		
Pearson correlation	−0.441	−0.610
*P* level	0.202	0.061
Lp(a) at baseline		
Pearson correlation	0.002	−0.165
*P* level	0.995	0.649

a *P* values were determined by two-tailed test. Abbreviations: ApoA1, apolipoprotein A-I; ApoB, apolipoprotein B; Lp(a), lipoprotein (a).

b There was a significant correlation between LDL and IL-6 at 24 h after LPS infusion. HDL and ApoA1 show significant correlations with IL-6 at both 360 min and 24 h after LPS infusion. For PCSK9 as well as ApoB, there was no significant correlation with markers of inflammation. All baseline values were measured before LPS administration.

### Lipoprotein response to inflammation.

There was a small, statistically nonsignificant difference in HDL as well as LDL levels at baseline between the two study days, with HDL mean values of 42.8 (placebo) versus 46.1 (LPS) mg/dl and LDL mean values of 105 (placebo) versus 114.3 (LPS) mg/dl (*P* = 0.175 and 0.170, respectively). Thus, for statistical calculations regarding LDL, HDL, ApoA1, and ApoB, values were corrected for the respective baseline value using a ratio to the baseline.

**FIG 3:**
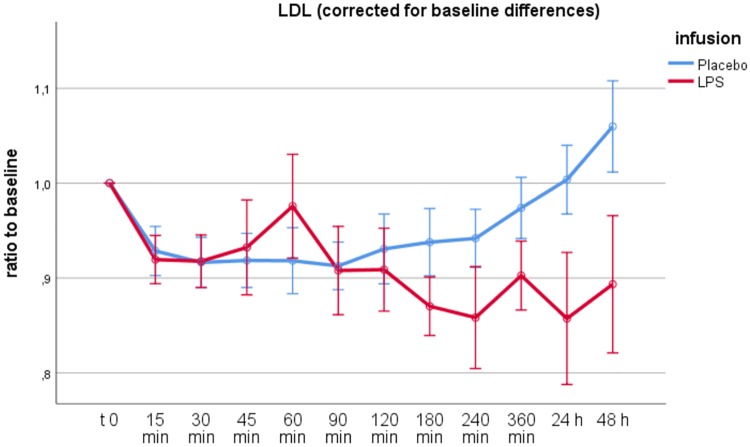
RM-ANOVA for LDL. The difference in LDL plasma concentrations between placebo and LPS administration was statistically significant (*P* < 0.001 using the Greenhouse-Geisser correction). Time points are shown on the abscissa, the ratio of LDL values to baseline is shown on the ordinate. Error bars depict 95% confidence intervals. Of note, there was a distinct peak in LDL levels 60 min after LPS administration. This relative elevation of LDL levels following LPS infusion was not statistically significant after correction for baseline difference (*P* = 0.065 using the Greenhouse-Geisser correction).

Following the administration of LPS, baseline-corrected plasma concentrations of LDL significantly differed from values after placebo administration, with LDL decreasing after LPS administration, especially after 90 min following infusion (*P* < 0.001) (Fig. 3). Of note, there was a distinct peak in LDL levels 60 min after LPS administration, which was not statistically significant when calculated by RM-ANOVA up to 90 min after infusion (*P* = 0.065).

While there was a significant difference in corrected ApoA1 levels (*P* = 0.003), there was no significant difference in corrected HDL (*P* = 0.073) (see Table S1 in the supplemental material) and ApoB (*P* = 0.267) values using the Greenhouse-Geisser correction of RM-ANOVA. Similar to LDL, there was a relative, statistically nonsignificant peak in HDL levels 60 min after LPS administration (*P* = 0.175).

### Lipoprotein plasma concentrations and inflammatory response.

**FIG 4:**
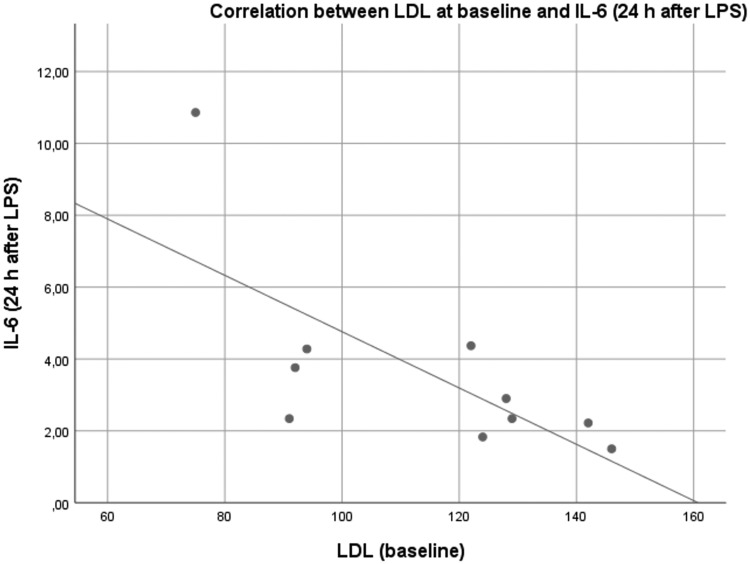
Scatter plot depicting the negative correlation between LDL levels at baseline and IL-6 levels 24 h after the administration of LPS. This negative correlation was statistically significant (Pearson coefficient of correlation = −0.699; *P* = 0.024).

Furthermore, we analyzed whether there was a correlation between plasma concentrations of different lipoproteins at baseline and inflammatory response. As shown in Table 1, plasma concentrations of HDL and ApoA1 at baseline correlated negatively with IL-6 at 360 min and 24 h after LPS administration. For LDL, this negative correlation was weaker and only significant for IL-6 levels 24 h after LPS administration (Table 1, Fig. 4, and Fig. S1 to S5). There was no significant correlation between ApoB or Lp(a) at baseline and markers of inflammation.

Due to these correlations of HDL and LDL at baseline with markers of inflammation, we further analyzed whether there was a correlation between the course of HDL and LDL following LPS infusion with markers of inflammation. For this purpose, the above-mentioned ratio of LDL and HDL values at a given time point after LPS infusion to baseline was divided by the respective ratio at this time point after placebo administration (see the supplemental material). This new parameter was calculated to illustrate relative changes of the two parameters at a given time point following LPS administration compared to placebo in a single parameter and was termed the change ratio.

The degree of the decrease of neither LDL nor HDL correlated with either marker of inflammation, as shown in Table 2. However, the relative peak in LDL levels as well as in HDL levels, as calculated by the change ratio at 60 min, correlated significantly with IL-6 levels at 360 min after LPS (*P* = 0.028 for LDL and *P* = 0.034 for HDL). The relative LDL peak at 60 min also correlated with IL-6 at 180 min after LPS (*P* = 0.031), as well as with CRP levels at 360 min (*P* = 0.049), 24 h (*P* = 0.012) and 48 h (*P* = 0.022) after LPS (Table 2 and Table S3 and Fig. S6 and S7).

**TABLE 2:** Correlations between inflammatory markers and the course of LDL/HDL as calculated by the change ratio^*a*^

Change ratio^*b*^	Value for:	
	IL-6 360 min after LPS	CRP 24 h after LPS
LDL		
60 min		
Pearson correlation	0.686	0.755
*P* level	0.028	0.012
240 min		
Pearson correlation	−0.329	−0.328
*P* level	0.354	0.354
24 h		
Pearson correlation	−0.353	−0.380
*P* level	0.316	0.279
HDL		
60 min		
Pearson correlation	0.671	0.612
*P* level	0.034	0.060
240 min		
Pearson correlation	−0.176	−0.256
*P* level	0.627	0.475
24 h		
Pearson correlation	0.069	−0.185
*P* level	0.849	0.609

a To illustrate the course of LDL and HDL levels after LPS administration relative to placebo, a change ratio was calculated by dividing the ratio of a value at a given time point after LPS administration to baseline by the same ratio following placebo. The degree of the decrease of neither LDL nor HDL, as calculated by the change ratio, correlated with either marker of inflammation. The change ratio at 60 min as a calculated number depicting the relative, nonsignificant peak of LDL and HDL at this time point correlated significantly with IL-6 levels at 360 min after LPS. In the case of LDL, there was also a significant correlation with IL-6 at 180 min (*P* = 0.031) as well as with CRP levels at 360 min (*P* = 0.049), 24 h (*P* = 0.012), and 48 h (*P* = 0.022) after LPS infusion. All correlations between inflammatory markers and change ratios at 60 min after LPS infusion were positive.

b *P* values were determined by two-tailed test.

## DISCUSSION

The human endotoxin model was successfully performed in this study in all 10 participants, with elevations of IL-6 and CRP after LPS infusion, as expected and previously published by other groups (24, 25). The decrease of PCSK9 plasma concentrations under fasting conditions observed after LPS and placebo infusion in our study is in accordance with the previously published diurnal variation of PCSK9 plasma concentrations (26).

### PCSK9 response in infection.

In our study of experimental human inflammation, there was no significant difference in PCSK9 plasma concentrations after the infusion of bacterial endotoxin compared to the placebo (Fig. 2). Moreover, there was no significant correlation between PCSK9 levels and any marker of inflammation. This stands in contrast to the hypotheses drawn from previously published *in vitro* data and clinical data in human sepsis (10, 13–15).

PCSK9 levels have been reported to be elevated in the early phase of sepsis and to be correlated with complications (15). Using immortalized human hepatocytes, it was reported that high concentrations of PCSK9 directly suppress uptake of lipopolysaccharide by hepatocytes and, thus, reduce endotoxin clearance. Thus, deficiency of PCSK9 seems to be beneficial in infection and sepsis due to improved endotoxin clearance (10, 27).

The pathophysiological purpose of this previously suspected elevation of PCSK9 in inflammation is largely unclear, even more so regarding the proposed negative effects of PCSK9 in this setting (10). Moreover, LDL levels were reported to be decreased in critically ill patients and severe infections (5, 6), although elevated levels of PCSK9 should, in theory, increase levels of LDL cholesterol. Thus, it has been suggested that the propagated increase of PCSK9 plasma concentrations in sepsis is a secondary effect following hypocholesterolemia after an inflammatory stimulus (8).

Our study is the first to evaluate PCSK9 response to experimental inflammation by LPS infusion in healthy probands. Previously published data reporting elevations of PCSK9 in infection have mostly derived from animal models (13, 14) and, in a clinical setting without a direct control cohort within the study, among septic patients upon presentation at the emergency department (15). Although measuring parameters in infection in a clinical cohort can identify important correlations in a real-life setting, it is impossible to know the exact onset of the inflammatory stimulus and the actual state of endotoxin clearance. This outlines the advantages of an experimental model, which may allow investigation of the underlying pathophysiological processes in greater detail.

Another reason for the disparity from previous data may be the rather brief presence of LPS in the bloodstream after bolus injection, in contrast to the prolonged presence of endotoxins in clinical sepsis as well as the sufficient capacity of young and healthy subjects to neutralize the limited amount of infused LPS. This inflammatory stimulus or the LDL decrease observed in our study may not have been sufficiently pronounced to trigger PCSK9 elevation.

Our results do not rule out a possible upregulation of PCSK9 in longer or very severe states of inflammation, such as sepsis. However, our data do not support a central role of PCSK9 in the human immune response to short inflammatory stimuli, as simulated in this experimental model.

### Lipoprotein response in inflammation.

The significant negative correlation between both HDL and ApoA1 at baseline and markers of inflammation after LPS infusion (Table 1) matches very well with existing data on this correlation and knowledge of the protective effects of HDL in inflammation (28), suggesting the validity of our data. In our study, we additionally found a positive correlation between LDL levels at baseline and inflammatory response (Fig. 4) that is, to our knowledge, yet unknown. This outlines the importance of HDL as well as LDL in the innate immune response to infectious stimuli and matches very well with population-based studies, in which associations between low LDL plasma concentrations and all-cause mortality in elderly populations have been shown (29, 30). This inverse correlation between LDL and risk of mortality in the elderly may be due to increased susceptibility to acute fatal diseases, such as infections.

In contrast to previous studies identifying hypocholesterolemia as a distinct negative acute-phase marker associated with complications and mortality (1–6), we found a rather more complex LDL response to inflammation in this setting. As expected, LDL levels decreased after LPS infusion, with a significant difference between the two study days (Fig. 3). However, this relative decrease only occurred after 90 min following LPS infusion. Over the first 90 min following LPS infusion, we observed a relative increase of LDL as well as HDL levels with a peak at 60 min after LPS infusion, although this short relative increase was not statistically significant after correction for baseline differences between the two study days.

Furthermore, our data indicate that the individuals with the highest inflammatory response to LPS infusion showed the highest relative LDL and HDL increase, since this relative difference, as expressed by the calculated change ratio, correlated with IL-6 and CRP levels (Table 2; also see Table S3 and Fig. S6 and S7 in the supplemental material). Interestingly, the extent of LDL decrease following LPS did not correlate with the degree of inflammation.

Naturally, the results regarding the dynamics of LDL over the first 90 min, as well as their correlation with the degree of inflammation, must be interpreted with caution due to the small number of subjects and other limitations based on the experimental setting of our study. However, these results are interesting, since such data can only be obtained from experimental studies like this, and such short periods of time following an inflammatory onset can hardly be investigated in a clinical setting, where the exact onset of inflammatory stimuli is usually impossible to know. It is known that lipoproteins play an important role in infection by binding and neutralizing pathogen lipids such as LPS. Considering the correlation between relative short-term upregulation of LDL following LPS with the degree of inflammation, it can be hypothesized that rapid upregulation of lipoproteins in infection reflects a defense mechanism against infectious antigen load to prevent excessive inflammatory processes and dysregulation of the immune response. On the other hand, it may also be interpreted that excessive short-term upregulation causes increased inflammatory responses. However, this remains mere speculation, for whether this nonsignificant short-term upregulation reflects a beneficial physiological response cannot be answered by our data.

While our data suggest that there are mechanisms raising LDL plasma concentrations in the very early phase of infections and that this rise correlates with the degree of inflammation, this effect of LDL recovery in inflammation is entirely independent of PCSK9.

### Conclusions.

Our data indicate that LDL response in the earliest phase of infections is more complex than previously thought and that LDL as well as HDL levels at baseline correlate negatively with inflammatory response. Although PCSK9 has been reported to be induced in animal models of inflammation as well as in human sepsis, our experimental data do not support this theory of PCSK9 augmentation in infection and inflammation, and no correlation between PCSK9 levels and inflammatory response could be observed.

## MATERIALS AND METHODS

The study was approved by the local research ethics committee (Institutional Review Board of the St. John of God Hospital Linz) and the ethics committee of the Medical University of Vienna. Informed consent was obtained orally and in writing from each subject before enrollment in the study.

### Protocol.

The study was performed as a prospective, single-blinded, randomized, placebo-controlled cross-over study. In total, ten healthy nonsmoking male subjects, aged 18 to 40 years, without any notable history of illness were included after a prescreening examination, which included a physical examination, routine laboratory testing, and an electrocardiogram.

On 2 different study days, separated by a washout period of at least 2 weeks, these volunteers received bacterial endotoxin (intravenous injection of 2 ng/kg of body weight national reference bacterial endotoxin over 5 min together with 0.9% saline during 90 min [200 ml/h]) or saline alone as a placebo (intravenous injection of 0.9% saline [5 ml] over 5 min together with 0.9% saline during 90 min [200 ml/h]) on the other day in a random order and single-blinded manner.

Subjects were studied at 8:00 a.m. after an overnight fast. Participants were asked to refrain from caffeine-containing beverages 24 h before as well as throughout the study day. Subjects were allowed to eat after completion of the respective study day (6 h after infusion) and were allowed to drink nonsparkling mineral water during the study day.

Intravenous catheters (B-Braun) were inserted into a vein on each arm (infusion line and sampling line). During the study, subjects rested in a supine position and were monitored continuously (electrocardiogram, heart rate, noninvasive blood pressure, and temperature).

U.S. standard reference endotoxin (lot 94332B1) was obtained from the Investigational Drug Management at the National Institutes of Health (NIH), Bethesda, Maryland. The purified lipopolysaccharide was prepared from Escherichia coli O113 and vialed under good manufacturing practice guidelines. Endotoxin was supplied in vials as a sterile, white, lyophilized powder, with each vial containing 10,000 endotoxin units (1 μg). Before infusion, endotoxin was reconstituted with sterile water and prepared according to the recommendations of the manufacturer.

### Blood sampling, laboratory measurements, and statistical analysis.

Blood samples for the measurement of PCSK9 and other parameters were taken at multiple points. The first blood sample was taken after the placement of the intravenous sampling line. After infusion of LPS or placebo, repetitive sampling was performed after 15, 30, 45, 60, 90, 120, 180, 240, and 360 min as well as 24 h and 48 h after infusion. The last two samples of blood were taken at 8:00 a.m. after overnight fasting.

Using VACUETTE polyethylene terephthalate glycol blood collection tubes (Greiner Bio-One), EDTA and lithium-heparin anticoagulated blood were collected. IL-6, C-reactive protein (CRP), HDL cholesterol, LDL cholesterol, apolipoprotein A1 (ApoA1), and ApoB were quantified within 2 h of blood collection from all study participants in lithium-heparin plasma. IL-6 was determined with a chemiluminescent microparticle immunoassay on a Cobas e411 Hitachi (Roche Diagnostics). CRP, HDL cholesterol, and LDL cholesterol were directly measured with standard assays on an Architect c16000 analyzer (Abbott Diagnostics). ApoA1 and ApoB were measured with standard assays on the BN-Prospec system (Siemens Healthcare Diagnostics).

EDTA plasma aliquots were stored at −80°C and subsequently used for determination of PCSK9 plasma concentrations, which were measured in one batch approximately 1 month after the recruitment period on a BEP 2000 instrument (Siemens Healthcare Diagnostics) with a quantitative PCSK9 sandwich immunoassay (Quantikine ELISA; R&D Systems) according to the manufacturer’s instructions.

To evaluate the precision of the PCSK9 assay in our laboratory, we performed a replication study according to the Clinical and Laboratory Standards Institute (CLSI; formerly NCCLS) guideline EP5-A. Two pooled patient plasma samples were aliquoted into twenty 1.5-ml plastic tubes for each concentration level and frozen at −80°C. We analyzed these samples in duplicate in one run per day for 20 days on a single BEP 2000 instrument. Within-run and total analytical imprecision (CVA) was calculated with the CLSI single-run precision evaluation test. The PCSK9 assay had a within-run CVA of 4.7% and a total CVA of 5.9% at a mean concentration of 156 ng/ml and a within-run CVA of 4.5% and a total CVA of 5.3% at a mean concentration of 226 ng/ml.

Statistical analysis was performed using IBM SPSS Statistics 25. Statistical tests included paired *t* tests and repeated-measures analysis of variance (RM-ANOVA). When sphericity could not be assumed according to Mauchly’s test of sphericity, the Greenhouse-Geisser correction was used.

## Supplementary Material

Supplemental file 1
