# Supplementary material for: Proprotein Convertase Subtilisin/Kexin Type 9 (PCSK9) Is Not Induced in Artificial Human Inflammation and Is Not Correlated with Inflammatory Response
Source: Infect Immun. 2020 Feb 20;88(3):e00842-19. doi: 10.1128/IAI.00842-19 (PMC7035937; doi:10.1128/IAI.00842-19)
Supplement: Supplemental file 1 [file zii999093007s1.pdf]

Online Supplement - Content

Differences at baseline between study days 2

HDL ratio ANOVA (corrected for baseline difference) – response to LPS infusion 3

Correlation between lipoproteins at baseline and markers of inflammation (IL-6 and CRP) after LPS infusion 4

ApoA1 4

HDL 6

“Change Ratio” calculation 7

Correlations – LDL peak (“change ratio” 60 min after LPS) and inflammatory markers 8

Pearson Correlation (table) 8

Graphic illustration (Scatter Plot) 9

Differences at baseline between study days

	unit	Placebo – baseline	LPS - baseline	p value
LDL	mg/dL	105	114	p = 0.170
HDL	mg/dL	42.8	46.1	p = 0.175
ApoA1	mg/dL	135.8	141.2	p = 0.297
ApoB	mg/dL	73.1	77.9	p = 0.178
Lp(a)	mg/dL	16.0	15.4	p = 0.805
PCSK9	ng/mL	205.2	203.7	p = 0.920
CRP	mg/dL	0.10	0.11	p = 0.840
IL-6	pg/ml	2.7	3.1	p = 0.528

Table S1 baseline differences between the two study days

None of the parameters shown in Table S1 differed significantly at baseline between the two study days. Nevertheless, for ANOVA-calculations, values of LDL, HDL, ApoA1 and ApoB were corrected for baseline values of the respective study day using a ratio to baseline.

Baseline differences of PCSK9, IL-6, CRP and Lp(a) were considered insignificant.

HDL ratio ANOVA (corrected for baseline difference) – response to LPS infusion

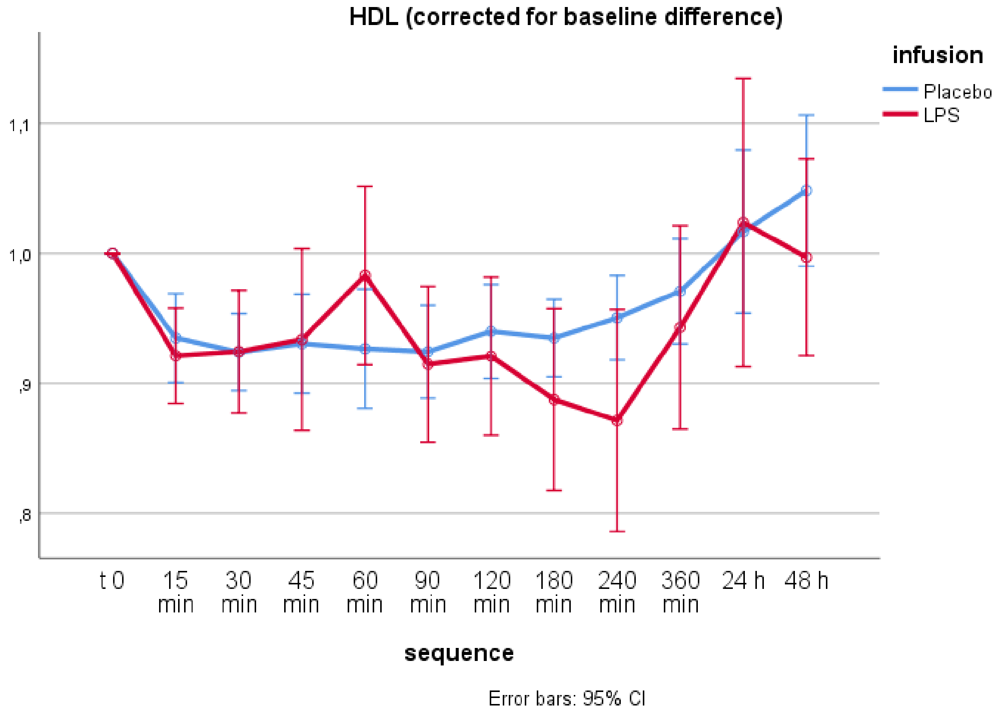

Table S2 RM-ANOVA – HDL

The difference in HDL - plasma concentrations between Placebo and LPS administration was not statistically significant (p = 0.073 using the Greenhouse-Geisser correction).

Time points are shown on the abscissa, the ratio of HDL values to baseline is shown on the ordinate. Error bars depict 95% confidence interval.

Of note, there was a distinct peak in HDL levels 60 minutes after LPS administration. This relative elevation of HDL levels following LPS infusion was not statistically significant after correction for baseline (p = 0.175 using the Greenhouse-Geisser correction).

Correlation between lipoproteins at baseline and markers of inflammation (IL-6 and CRP) after LPS infusion

In this online supplement, correlations between ApoA1 as well as HDL at baseline with inflammatory markers are depicted graphically. The correlation between LDL at baseline with IL-6 at 24 hours is shown in the manuscript.

ApoA1

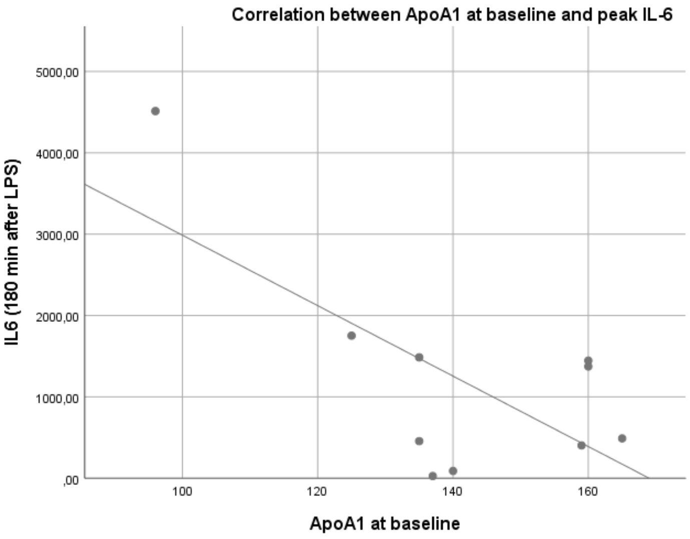

Figure S1 Scatter Plot depicting the negative correlation between ApoA1 levels at baseline and peak IL-6 levels (180 minutes after the administration of LPS).
This negative correlation was statistically significant (Pearson coefficient of correlation = -0,689; p = 0.028)

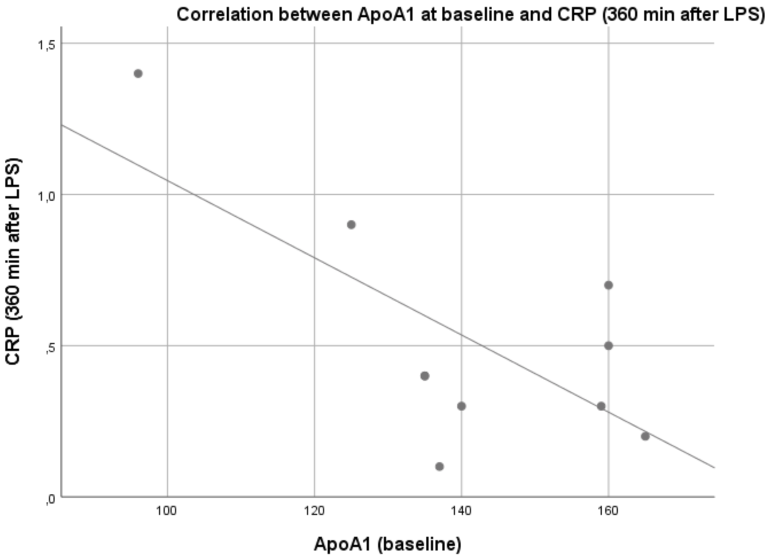

figure S2 Scatter Plot depicting the negative correlation between ApoA1 levels at baseline and CRP levels 360 minutes after the administration of LPS.
This negative correlation was statistically significant (Pearson coefficient of correlation = -,692; p = ,026)

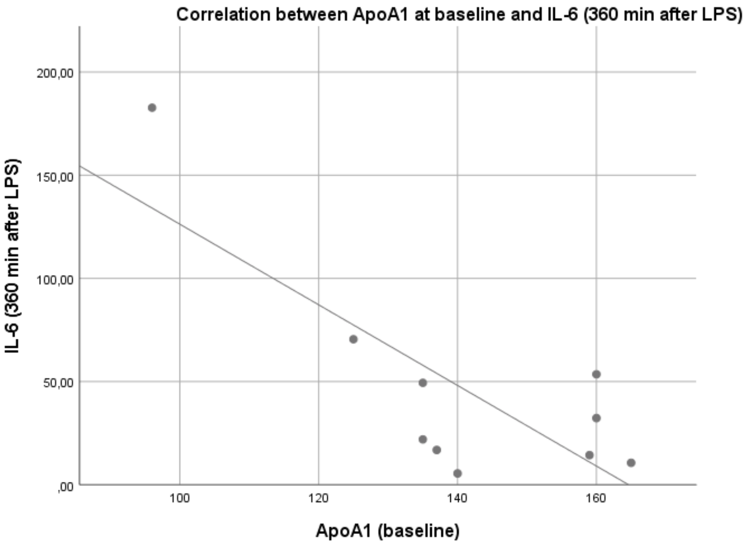

figure S3 Scatter Plot depicting the negative correlation between ApoA1 levels at baseline and IL-6 levels 360 minutes after the administration of LPS.

This negative correlation was statistically significant (Pearson coefficient of correlation = -,783; p = 0.007)

HDL

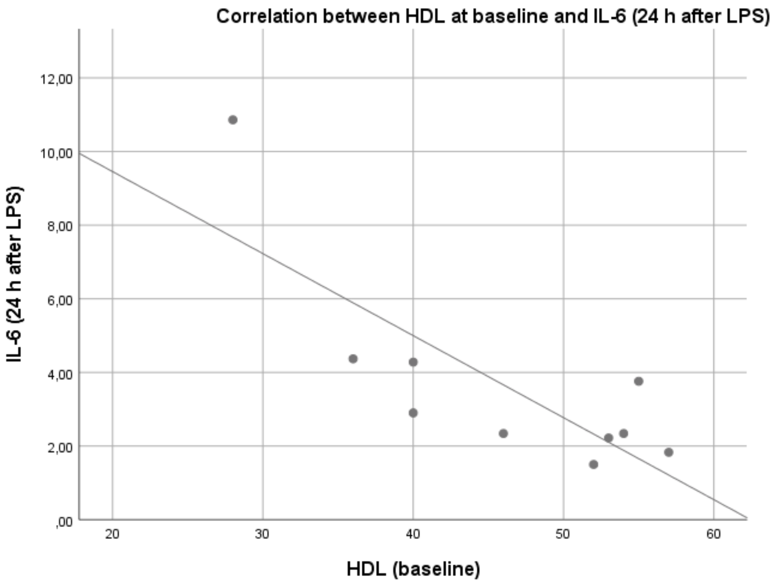

figure S4 Scatter Plot depicting the negative correlation between HDL levels at baseline and IL-6 levels 24 hours after the administration of LPS.
This negative correlation was statistically significant (Pearson coefficient of correlation = -,793; p = 0.006)

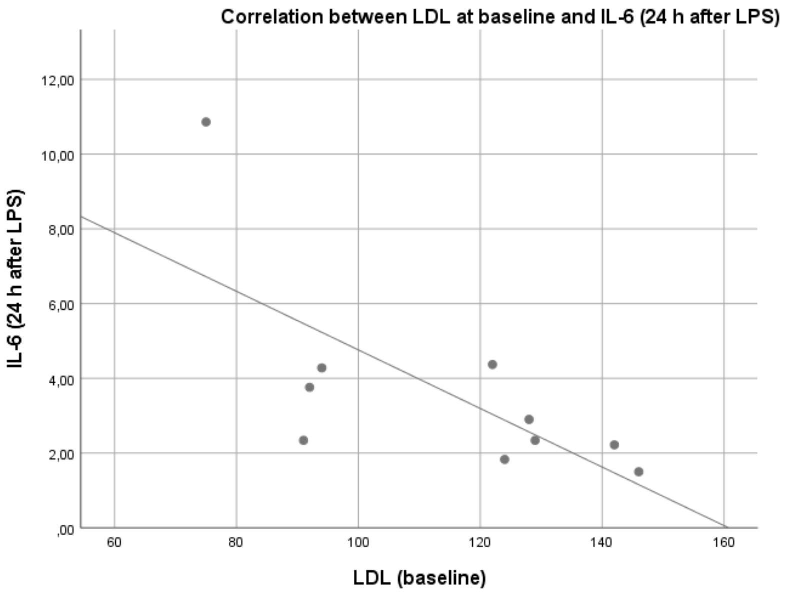

figure S5 Scatter Plot depicting the negative correlation between LDL levels at baseline and IL-6 levels 24 hours after the administration of LPS.
This negative correlation was statistically significant (Pearson coefficient of correlation = -,699; p = ,024)

“Change Ratio” calculation

To mathematically illustrate the course of LDL and HDL levels after LPS administration relative to placebo, a “change ratio” was calculated by dividing the ratio of a value at a given time point to baseline by the same ratio following placebo.

For example, to calculate the “change ratio” for LDL at 60 minutes after infusion, the following formula was used:

$$\text{Change ratio LDL (t 60)} = \frac{\frac{LDL\ (LPS\ t60)}{LDL\ (LPS\ t0)}}{\frac{LDL\ (Placebo\ t60)}{LDL\ (Placebo\ t0)}}$$

This “change ratio” was then used to calculate correlations of relative changes at a given time point with markers of inflammation to evaluate, whether the extent of inflammatory response correlates with the LDL/HDL response to inflammation.

Correlations – LDL peak (“change ratio” 60 min after LPS) and inflammatory markers

Pearson Correlation (table)

Correlations – LDL peak (“change ratio” 60 min after LPS) and inflammatory markers

		CRP[⁞] 360 min after LPS	CRP[⁞] 24 h after LPS	CRP[⁞] 48 h after LPS	IL-6* 180 min after LPS	IL-6* 360 min after LPS
**“change ratio” LDL[||] at 60 minutes**	Pearson Correlation	**.633**	**.755**	**.707**	**.678**	**.686**
	p-level (2-tailed)	**.049**	**.012**	**.022**	**.031**	**.028**
**“change ratio” HDL[‡] at 60 minutes**	Pearson Correlation	.604	.612	.551	.572	**.671**
	p-level (2-tailed)	.064	.060	.099	.084	**.034**

Table S3 Correlations between inflammatory markers and the course of LDL/HDL as calculated by the“change ratio”

To illustrate the course of LDL and HDL levels after LPS administration relative to placebo, a “change ratio” was calculated by dividing the ratio of a value at a given time point after LPS administration to baseline by the same ratio following placebo.

The LDL “change ratio” at 60 minutes after LPS infusion as a calculated number depicting the relative, non-significant peak of LDL at this time point, correlated significantly with CRP and IL-6 levels. The HDL “change ratio” correlated significantly with IL-6 at 360 min after LPS.
All correlations between inflammatory markers and “change ratios” at 60 minutes after LPS infusion were positive.

*Abbreviations: *: interleukin-6; ‡: High-density lipoprotein; ||: Low-density lipoprotein; ⁞: C-reactive protein.*

Graphic illustration (Scatter Plot)

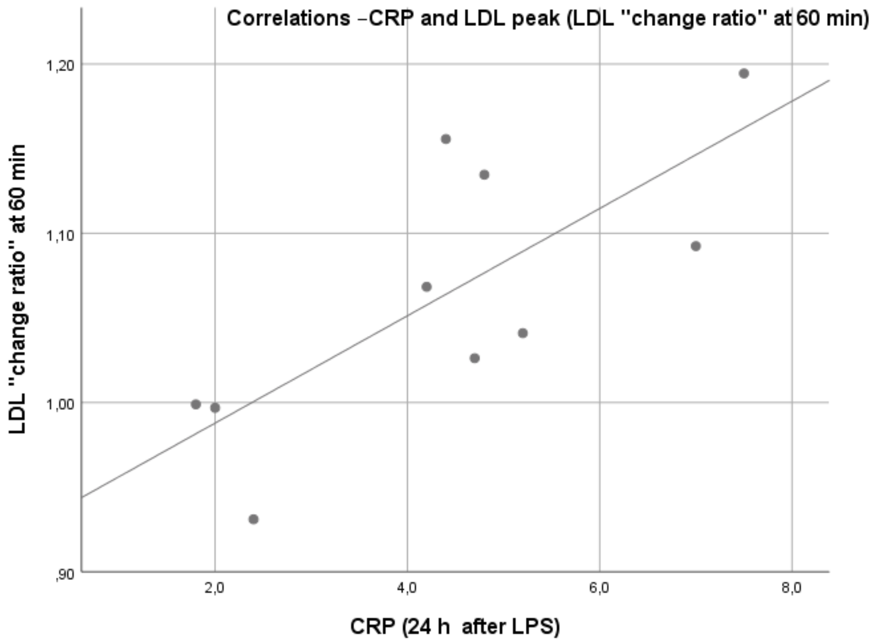

Figure S6 *Scatter Plot depicting the negative correlation between the non-significant LDL peak, as calculated by the "change ratio" at 60 min after LPS infusion, and CRP levels at 24 hours after LPS.*

This correlation was statistically significant (Pearson coefficient of correlation = 0.755; p = 0.12)

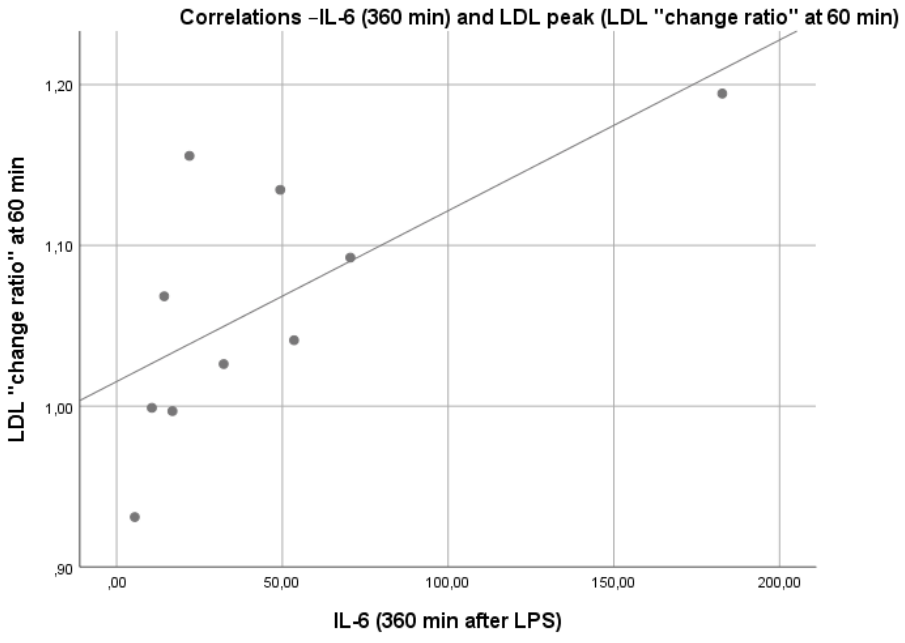

Figure S7 *Scatter Plot depicting the negative correlation between the non-significant LDL peak, as calculated by the "change ratio" at 60 min after LPS infusion, and CRP levels at 24 hours after LPS.*

This correlation was statistically significant (Pearson coefficient of correlation = .686; p = .028)
